# Beyond the clinical context: the process of losing oneself living with Huntington’s disease

**DOI:** 10.1186/s13023-022-02330-9

**Published:** 2022-05-07

**Authors:** Luz-Estella Varela, María-Mercedes Arias, María-Antonia Martorell-Poveda, Clara V. Giraldo, Rosa A. Estrada-Acuña

**Affiliations:** 1 Nursing Faculty, University of Antioquia, Medellín, Colombia; 2 Nursing Faculty, Universidad Rovira I Virgili, Tarragona, Spain; 3 Bio3Science Research Network, Belonging to Geography and Urban Studies, Temple University, Philadelphia, USA

**Keywords:** Loss of autonomy, Physical dependence on another person, Loss of sense of self, Grounded theory, Neurodegenerative condition

## Abstract

**Background:**

People with Huntington's disease (HD) have increased functional and cognitive dependence. While numerous clinical, genetic, and therapeutic management studies have been carried out, few studies have investigated the disease from the personal experience and the context of people living with HD. To better serve these patients, our purpose is to understand, from the perspective of the patient and their families, how people with HD cope with their daily lives outside the clinical setting.

**Methods:**

Thirty-three affected or at-risk people participated in this study. Participants were interviewed at their homes on distinct occasions during a family visit. We analyzed the data using Grounded Theory, which allowed us to understand how people live with the disease on their own terms.

**Results:**

Living with HD is a process that begins with acceptance or denial that one is at risk for the disease or, growing awareness of the condition due to motor, behavioral, and cognitive changes, and, finally, loss of autonomy with physical dependence on another person, and loss of sense of self and family.

**Conclusion:**

While the daily life of patients before disease onset was characterized by physical and mental/cognitive independence, with HD they become increasingly trapped in their bodies, and their complications are due to the lack of effective curable therapy.

**Supplementary Information:**

The online version contains supplementary material available at 10.1186/s13023-022-02330-9.

## Background

Huntington’s disease (HD) is a rare [1] neurodegenerative condition transmitted genetically and developed during young adulthood, although it can have latent forms in elderly adults [2]. It has severe consequences due to the bodily and mental compromise it produces and the care the affected person needs. The symptoms consist of a triad of behavioral, motor, and cognitive alterations, leading to death from 10 to 20 years.

The daily life of people with HD involves a series of complex situations that are central to the current study. The first is the genetic dimension. HD is passed from parent to child through genes. Then if one of the parents has the disease, their children, regardless of gender, will have a 50% chance of developing it. The cause of this neurodegenerative disease is a mutation in the gene called huntingtin (HTT), located on chromosome 4 of the human being. The normal HTT gene contains a sequence of up to 36 Cytosine-Adenine-Guanine (CAG) amino acids, but when the number of CAG repeats increases, especially above 40, the HTT gene is considered mutated. This mutation leads to the formation of an abnormal protein that accumulates inside the body´s cells, this causing a triad of alterations [3].

The second dimension is psychological, consisting, on one side of the uncertainty in developing the disease upon learning that one belongs to a risk group. Also, people suffering from HD have a higher rate of suicide than the general population [4].

Inheriting a condition poses personal, family, and social dimensions that have to do with the way the disease interrupts life-HD has a higher initiation frequency between 35 and 55 years of age [2], a stage during which society expects us to be most productive, such as having a family and becoming economically independent [5–7].

A third economic dimension derives from these conditions, given that HD a disease with catastrophic consequences due to the “high technical complexity in its management, high cost, low occurrence, and low cost-effectiveness in its treatment” [8], according to Legislation 972 of 2005 [9].

A fourth social dimension focuses on the stigma endured by people who suffer from the disease, particularly those whose body changes are more noticeable, especially involuntary movements. Goffman proposes that stigma is a situation when a disabled individual does not have full social acceptance [10]. A connected administrative and legislation dimension is related to the fact that HD is a rare or orphan disease. According to Colombian legislation, HD is a condition that affects less than 1 in 5000 people [11]. Current legislation in Colombia (Legislation 1438 of 2011) [12] ostensibly protects the health of people suffering from rare diseases, but, for people with HD, the fundamental right to health has been violated because such legislation is not executed in ways that impact the everyday life of people with HD.

A fifth, epidemiological dimension, entails the significance of migration from Venezuela across the eastern Colombian border, which is of concern [13], especially around Lake Maracaibo. This area has the highest prevalence of HD in the world [14]. Lack of trustworthy sources about the health status of the immigrant population across the region hinders the clarity of systematic, periodic, and rigorous records of HD.

From a social-sanitary point of view, HD is a little-known rare disease. Technical guidelines for care and strategies for different phases of the disease are poorly known by health personnel. They lack information to educate carriers and people at risk, including adolescents, parents, and co-workers. Also, they need more information to advise people with HD about retirement plans and how to care for themselves when working.

The daily life of people with HD takes place amid complex conditions. To address this, we proposed to answer the question: How do people with HD live in the Colombian context? This study aimed to analyze how HD affects the daily life of people at home and, specifically, to analyze the process of loss of sense of self.

## Method

### Study design

Our study included qualitative analysis of 40 interviews carried out with 33 patients at risk or diagnosed with HD. Qualitative methodologies are better suited to studies of phenomena related to the understanding of experiences [15]. Qualitative methods allowed us to understand the everyday life of people with Huntington in their own terms. For the data analysis, we used specific procedures following the methodology of Grounded Theory. We implemented open, axial, and selective coding techniques for analysis, and we identified a core category and subcategories with specific properties [16].

### Participants

The study had 33 participants. In Colombia, due to the socio-economic costs of genetic tests for the diagnosis of HD, diagnosis is usually made based on the presence of the characteristic symptoms of the disease and family history. Consequently, our inclusion criteria were: Adult individuals in Colombia (18 years old and older), who are at risk of HD owing to family history or already diagnosed with HD through genetic testing or characteristic symptoms, with cognitive and language capacity to answer the interview questions. Or, a person who is a caregiver of someone in the final stage of HD. Diseased persons living alone and lacking cognitive and language capacity to participate in the interview were excluded from the survey. The participants came from different regions of Colombia, South America: Medellín, Bogotá, Santa Marta, and Chocó, principally Juan de Acosta on the Atlantic. The region around Juan de Acosta is especially significant as it has the second highest number of affected people after Venezuela [14]. The 33 participants included 19 men and 14 women between 22 and 63 years of age. The average age was 42 ± 12 years. Five participants were at risk, five in anosognosia, six in the initial phase, 14 in the intermediate phase, and three in the late phase. At least 27 participants depended economically on their family, six received a minimum wage or less, and two were homeless. In the healthcare system, 23 participants depended on subsidized coverage, the other ten paid for their healthcare. The Neuroscience Group in Antioquia and the associations of the regions facilitated contact with the participants.

### Data collection

To collect the data, we used semi-structured interviews with a guide previously prepared by the researchers (Additional file 1). Before the interviews, we agreed on a date and time with the participants. The interviews began with information on the duration of the interview, signing of the informed consent, and permission to record. No one denied their participation or withdrew from the process. All interviews were recorded and transcribed [17]. The participants were asked to express themselves freely. Family members of HD patients were present at all of the interviews. Most of the patients answered the questions. Relatives helped to complement answers, especially when the patients had cognitive impairment. We carried out the interviews following the data analysis process. We collected 22 interviews during the initial or open coding, 15 interviews during the axial coding, and three more to saturate the data in the selective coding phase. One interview was conducted virtually via Skype, and the others were face-to-face; three were conducted during a medical appointment, and the other 36 were in the natural place where the people lived. Seven of the participants had two interviews with the intention of complementing the information collected. Some questions included in the interview were: How did the disease spread to your family? Can you describe a typical day? Has your life changed now compared to before? How do you think other people perceive you? Have you thought about the future? How did you imagine the future? Each interview lasted an average of 60 min. The first author performed all of the interviews.

### Data analysis

We performed the data analysis using GT in three stages: open, axial, and selective coding. The open coding of data consisted of the careful reading of the transcripts of 22 interviews. Then we fragmented the data (the transcripts) into meaningful sentences and paragraphs. Each sentence or paragraph was assigned a "code" (Label that researchers assign to a significant paragraph or unit of analysis that caught our attention). At this stage, we created more than 1458 codes. Each code had initial memoranda—memos or notes with different levels of abstraction and depth made by the researcher during the analysis process [16]. The first categories emerged through the abstract grouping of codes that developed during this stage with their respective memoranda.

In the axial coding, we continued the analysis starting from the categories that emerged in the previous stage. At this stage, we included 15 new interviews (transcribed). The objective of these interviews was to enrich the concepts and to make the categories denser. The transcripts were fragmented, and each sentence or paragraph was assigned a “code.” At this stage, we created more than 995 codes. Each code was compared with paragraph, or phrase, code, and memo that made up the categories that emerged in the open coding. We managed to reaffirm some previous categories, and new ones also emerged. In this part of the analysis, we submitted each category to questions such as What is happening? Why? Where? How? When does it happen? And to Whom is it happening? The answer to such questions allowed us to generate new codes and memos to refine the categories. The analysis continued until categories developed, together with subcategories and properties [16].

For selective coding, we started from the categories and subcategories developed in the previous stage. We carried out a regrouping of the data, and a core category and four subcategories, with properties, emerged. At this stage, we included three new interviews (transcribed). Using these three interviews, it was possible to achieve theoretical saturation of the data, because they did not offer any additional properties in relation to those that already existed in the core category that emerged during axial coding. The diversity of characteristics among the participants facilitated the opportunity to discover variations between the concepts, to make the categories denser [16]. We shared the information found in the analysis with the participants to validate the findings with them. Finally, we constructed a written narrative with the new theory.

The study followed the recommendations of Resolution 8430 by the Colombian Ministry of Health [18] and followed the guidelines of the Helsinki Declaration [19]. The project was approved by the research ethics committee of the Faculty of Nursing at Universidad de Antioquia, Record Nº CEI-FE 2017-5 of 10 February 2017.

## Results

Based on the mentioned analysis, the core category that emerged was: “Loss of sense of self until death.” The theoretical development of the core category corresponds to a substantive theory about the daily life of people with Huntington's disease, which is related to the “allegory of the cave” that Plato describes in his text The Republic [20], but inversely. This myth describes those who inhabited the cave born as prisoners who could only see the shadows reflected on the wall by the bonfire. Those affected by HD were born free; upon initiation of the disease, they enter the cave, which limits, shortens, and reduces them, leaving them prisoners in their own body; from the outside, they only have the shadows, the memories. There is no exit from the cave; the only escape is death.

This theme emerged as the core category of our study because it was the one with the most intense relationships with the other categories constructed in the data analysis. The core category is accompanied by four subcategories: (a) context, (b) loss of sense of self, (c) relationship, and (d) daily life, each with its properties (Additional file 2). For this article, we will focus on the second subcategory, loss of sense of self, because to understand the process that people go through when they start to lose themselves in the course of the disease offers important clues to consider in the care of people with this pathology.

**Fig. 1:**
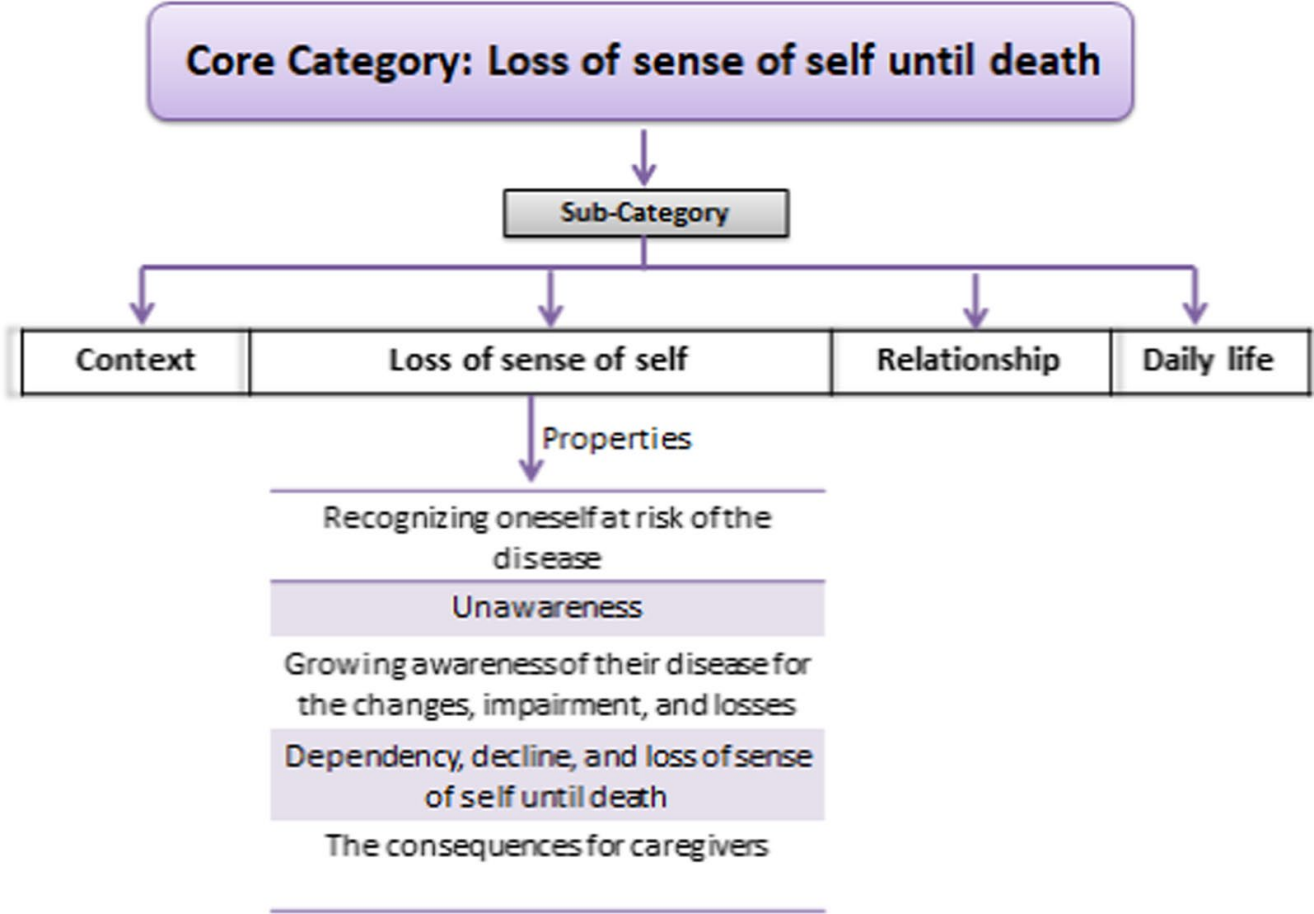
The fives properties of the subcategory loss of sense of self

The subcategory “loss of sense of self” has five properties: (1) Recognizing oneself as at risk of the disease, (2) Unawareness, (3) Growing awareness of the disease due to the changes, impairments, and losses. (4) Dependency, decline, and loss of sense of self until death. (5) Consequences for caregivers (Fig. 1). The first four properties indicated steps in the process of *Loss of sense of self*, and the last one shows the consequences to caregivers or families that live with a patient that suffers from HD.

To lose one’s sense of self is to stop being the person that was, and to be other than she/he/they recognized by themselves or their family or their previous social context. In this journey, ill people are exposed to stigma, above all, due to involuntary movements and behavior alterations. This state cannot be modified, and it only will cease with death.

See Additional file 2 for details of the core category, subcategories, and their properties.

### Recognizing oneself at risk of the disease

Being a person at risk, that is, being the descendant of a father or mother who had the disease or has the gene, is a reality faced by family members, parents, children, the partner or spouse, and other people like neighbors or friends. Some people know about their HD risk once they have already married, have had children, or have started specific work-life projects.

Recognizing oneself as at risk generates a state of concern or uncertainty that Mishel defines as the incapacity to grant defined values to objects or events, or not being able to predict with precision the outcome [21]. This uncertainty leads people to rethink about the convenience of having a genetic test to confirm the disease. If they have a diagnosis of the disease, they often fear that they will not find a way to deal with it and the consequences it will have on their family and social life. Therefore, some people who recognize themselves as at-risk prefer not to undergo genetic testing or to learn more about their disease. They decide to live day-to-day, and not think about the future, leaving everything in the “*hands of celestial beings,*” and not wanting to know more about the disease so as not to give force to negative thoughts or be restless. For others recognizing oneself at risk implies that the person begins to think about the possibility of taking the diagnostic test to make decisions, although there is fear of the correctness of the diagnosis.

I think that if your brain does not know that it exists… (It does not worry you), before then I did not know it existed; now I confront it because I know it exists. …But when I did not know it existed, I led a super normal life, nice. I had no problems, no day to depress me, to think if my finger moved, because thank God, it moves… and I get depressed, so imagine, so these are things that if I had not looked up in that internet, no, I would be happy in life. …do you understand me? Because I would not have known… because I think everything is in the mind.

Knowing the genetic status and confirming the disease brings about new concerns regarding whether or not to communicate the diagnosis to one’s partner, family, and social and work circles. When people give the news to their children about the condition of the disease and their own risk of getting it, it is not easy. Some parents even postpone telling their children until they are adults and have also had children. The patients who told others about their illness speak of discrimination not only at work but also by their partners.

When I married him, people asked me: Did you not know about the disease? I did know it, but there they were rejected; being such a nice man but there they were rejected many times and so it is so difficult for those people to get engaged, a sister of his got married, and it did not last a year, the husband found out about that disease and immediately left.

Another concern that a person at risk or with HD has is the duality of having or not having children. In the case of having them, they have the uncertainty that the disease could be inherited. Women report more concern about the conditions in which their children will live (who will care for them) when they are in the final phase of the disease or have died. Decisions about motherhood and fatherhood are individual or joint matters for the couple, and also have a cultural or religious component. Although patients receive recommendations about the risks of having children, it is they who decide. Opinions about having children are varied, some of them express the desire not to have them or consider adoption as an option, others know the risks they would have, and the most relevant motivation to have a child is that they would like to be cared for when they will get very ill.

It is a cluster of things, that is, the disease that let’s say, even if we (the grandparents) manage it, now it does not hurt as much… But the contour, their children and all those things… and with the uncertainty of knowing when… that thing is heartbreaking, you don’t know if they will inherit it… hopefully not.

Ah, it is so difficult. In any case, the children are the family of one, in the same way, imagine one with this disease and arriving alone, and the need for someone to be with.

### Unawareness

Clinically, anosognosia occurs when a person with brain dysfunction appears unaware of any impaired neurological or neuropsychological function, while this is evident to the physician and others [22]. In our participants, this condition does not allow people to have appropriate expectations about their disease because they cannot see themselves as affected. The incapacity to see oneself as affected we refer to as “unawareness.” Not being aware often explains why the person continues performing habitual activities, unpersuaded by the consequences of the risks to oneself and others, for example, the danger of driving an automobile or using substances that cause harm given their condition.

Unawareness is a situation experienced by many people with HD, although some do not go through it and their relatives recognize that they were conscious of the disease since the beginning. “Many of them are aware and know where they are going; they know what they have, where they are going, what is expected, what they would need”.

The sub-process of unawareness of their disease condition has variations, from *just not caring, having scarce conscience, medium conscience* to reaching *full conscience* of the disease that many do not reach. Not assuming the symptoms or recognizing the disease can delay the onset of the treatment or the support therapies of mobility and language, besides hindering adjustments to avoid risks under circumstances of their daily life, like public transportation or in the type of occupation they work.

*Just not caring* consists of someone wanting to persuade the diseased person of the changes, but they seem unconscious of their disease and do not grant importance to observations by others; said attitude may seem malicious or a strategy to deny the symptom.

My father has had it for about one year, only, it is only recent for him, he has this foot that moves. He is not aware that he moves it, and the hands, sometimes he goes like that... (shows the movement).

### Growing awareness of their disease due to the changes, impairment, and losses

“Growing awareness” is the state in which the person recognizes that their symptoms have changed their lives. Changes in their body and mind force them to abandon the roles that they perform. The involuntary choreic movements, consultations with the neurologist, and the medications prescribed persuade those affected to come to terms with reality.

Changes may or may not be visible at this stage. The most relevant visible changes may include: (a) Motor changes such as involuntary movements, changes in gait and language; (b) changes in behavior, conduct or personality. The least visible of changes is (c) cognitive impairment: memory, learning, orientation, and executive functions.

- ***Motor changes:*** On the one hand, there is an increase in involuntary movements in one part of the whole body, this being more frequent and intense than regular. On the other hand, mobility and muscle strength suffer a progressive deterioration until it affects activities of daily life—changes in gait, deterioration of balance and falls, so they need a dedicated caregiver.

These manifestations make HD a conspicuous or noticeable disease for other people, given that it compromises corporeity. The risks of falling and the difficulties in walking prompt external observers to the family group and friends to express judgment and criticism for the situation they suffer. Caregivers are also criticized for implementing unconventional actions to avoid accidents and falls. For example: placing the patient with HD on the floor on a mattress to avoid falling from the bed.

Some motor changes generate alteration in nutrition, it is the case of dysphagia,^1^ which makes it difficult for patients to swallow food, and causes drowning. Added to this are respiratory and behavioral problems. Some patients with HD tend to eat too fast, take large bites that make them more prone to choking; they have an unbridled desire to eat, which seems never to be satisfied.

In addition, involuntary esophagus movement and choreic movements hinder daily procedures such as brushing teeth or flossing that generates damaged teeth due to lack of hygiene, periodontal disease, plaque, and xerosis, or dry mouth, effects that can also be caused by neuroleptics, antidepressants, and anticholinergic medications. Consequently, many need gastrostomy feeding tubes, which require procedures, instruments, and preparation for this type of feeding; those who do not undergo this technique, finally, cannot swallow and die of hunger.

All the Huntington patients I have seen with this problem have mostly died of hunger. They reach a stage when they cannot eat or drink anything. They can’t even swallow liquids when they reach the fifth or sixth stage, at the end.

Adapting the toothbrush with a thick handle or using an electric toothbrush, blending food, or using thickeners can be alternatives used by the patient and their family members to deal with motor changes alterations.

(b) ***Changes of behavior, conduct or personality:*** Changes and deterioration occur gradually, constituting a long process that takes more than ten years. Individuals and their families defy circumstances to maintain functionality until the day things become untenable. Some behavioral changes reported by participants and their families range from carelessness in personal care, rush or slowness, obsessions, pretending to be a liar or unkind, use of incoherent expressions (due to speech disturbances). They also have hallucinations and difficulty in sleeping, with effects on their rest and the whole family. The emotional lability could be complemented by crying spells, sadness, depression, and even reach a state of madness.

She cries, we don’t know why … Sometimes she cries like a little girl and sometimes she cries for hours (long silence) uh, we suppose, we suppose it is because she does not accept the disease and feels totally frustrated by what is going on …with her life.

In addition, family members may learn of changes due to stubbornness, new habits of cursing or having manias, such as kleptomania, gambling, and persistent repetition of a word, phrase, or gesture [23].

She was sick, so she would start ah, ah, ah, her laughter, the disease made her become like that. Sometimes they have more manias than others”… “Well, there are days that he gets up happy, other days he is aggressive, uh, he chooses a theme, a theme and a theme and until the theme goes away….

Manias are also related to activities that they repeat during daily life, such as: bathing several times or not touching things with their hands, etc. Personal care is an aspect that humanizes the person suffering from HD. Impairment could consist of loss of hygiene and grooming ability, as well as loss of motivation.

Yes, she continued eating, doing everything on her own; she showered, changed her clothing. She ate well, went out… and so the impairment started until the moment that she no longer could… (she could do nothing)”… “She was a very exquisite woman, fine perfumes, nails well-polished, she liked being well dressed… and now she... (No).

The mood change may be constant, several of them become more docile, others more aggressive, and others continue as they were in this regard. Some manage to control their mood with medication. The process of coping with the changes of the sick family member can be less painful, understandable, and bearable when family members recognize the disease, unlike those who attribute it to a personal relationship style, such as being unpleasant or hostile.

Other visible changes related to behavior, conduct, and personality include the deterioration of voice, language, and oral communication. The communication deterioration process is gradual. The loss of language causes double anguish; for the patients because of not being able to express themselves and for their caregivers because of not understanding the patients. In this relationship, various communication strategies emerge, with or without success, depending on the interaction, patience, and dynamics of each family.

... Sadness because sometimes we did not understand what he was saying to us. He had to guess what he felt at that moment if he was hungry or thirsty because he no longer expressed himself very well. So that is a painful, painful death for them as well as for us.

(c) ***﻿Cognitive changes and other losses:*** Although less visible, these changes contribute to deteriorating relationships, sociability, and autonomy; HD is a disease of the body and mind. Relatives refer to a cognitive impairment that affects alertness, memory, and learning. Due to neuronal deterioration, people forget how to carry out typical daily activities such as shopping in-store, cooking, washing their clothes, and even dressing or bathing.

Cognitive alterations have consequences for academic and work performance because the patients forget how to perform some activities or do not remember the details involved. Patients with HD are prone to mistakes that may lead them to lose their job. Only a small number of our participants had a chance to retire. Most of our participants had informal employment, added to cognitive impairment, which implies other restrictions, such as not going out alone due to the risk of loss, not remembering instructions, or managing money in an insecure way.

She was a doctor and remembered that they told her: You have to get out of there because you can't keep making mistakes, and you can't imagine how the lawsuits are for doctors.

Orientation capacity also gradually deteriorates, which frequently causes people to get lost in the street, putting themselves at risk of suffering from an accident and even sexual abuse. When this happens, the family can understand the inability of patients with HD to care for themselves at this stage of the disease.

She would get out of the house, once she was grabbed and raped by a number of men, who managed to see what was being done… because she does not like being locked in but to go out.

Patients and family members notice the deterioration of what is known as recent memory or working memory. Some patients express the loss of ability to reading, and the capacity to learn something new. The loss of cognitive ability is shown by patients with HD slowly. When people with HD have impaired cognitive ability, they must make an effort every day to remember how to perform the most routine and typical activities such as taking a shower.

Since I got the disease, I forgot about reading (laughter)”… “One day the mind can be lucid and another day not”… “-At what time would he get up? He would normally get up incredibly early, at five or six, but with the disease, he would be more in bed, and it is harder to get up. -Would he shower and eat? Many hours, getting up, showering, having breakfast, three hours... like, like, before when he was not sick, he did that in 25 minutes, but in recent months no... Now it is three hours. That is why, he does everything very slowly, and everything is slow.

### Dependency, decline, and loss of sense of self until death

Dependence and the loss of the self until death is the state of loss of autonomy and the need to depend on another person, which leads the patient to be different from who he once was, someone who neither his family nor he himself recognizes. As already described, functional losses increase in intensity, lead to impairment, and increase dependency and the need for a caregiver. The sum of losses obligates to the need to give up – voluntarily or not –everything that once gave joy, including things like mobility, driving an automobile, or even smoking.

When they begin with the disease, they might perform some tasks, they do their things, but as they advance, they start losing the capacity to perform their tasks, so now they dedicate more time to sitting, to walking, to going out, but then they do nothing.

This cognitive impairment process and all the changes described lead to the loss of their sense of self, of their being, of their body image. People stop being what they were in the past and go on to appear as another person they themselves do not recognize. Their relatives miss the “old” version of them, as they state, *“He was a charming person, a gentleman”.* People with HD at the beginning of their lives have a free daily life, and because of the disease, they gradually lose their of sense of self, not recognizing who they were or how their life was before they developed the disease. As stated by Charmaz “people embark on a ship that takes them to a profound darkness, to the decline, and death” [24].

### The consequences for caregiver

The dependence of HD patients on their caregivers implies that they provide the patient with essential care such as keep them clean and fed. The caregiver is also a means for the HD patient to communicate with others, since caregivers learn by close interaction with the patient to interpret movements, gestures, and sounds, and to "translate" their needs and desires. Long-term care of a sick person with HD generates a series of consequences in families and the caregivers themselves that we can classify as economic, emotional, and physical.

**Economic** consequences occur because family members must leave their jobs to dedicate themselves to the specific care of the person with HD, this modifying family roles and causing a decrease in income. If the costs of the disease are high, coupled with the reduction in income families become even poorer, this affecting the acquisition of goods and services that are essential to their well-being.

In our study, the economic deficit of families is a relevant issue because before the disease appeared, many of them already reported insufficient financial status, so the disease worsens the situation. A higher family income enables direct caregiving, and placing relatives in the care of suitable professionals, which alleviates "the burden of care." Meanwhile, families with low income and without adequate support experience a substantial increase in the burden of caring for one of their members, this also increasing their physical and emotional exhaustion.

Sometimes, doctor, we do not have money, horrible, right, Dolly? For example, the day I went with Dolly, to this one, not for breakfast or anything ... I traveled disgusting (laughs), just as we left that way we came, no more than with mere passages. And I said: no, we are going to buy a little piece of bread, when we returned we made ourselves a black coffee, a black coffee with bread, that is what we practically had for lunch ... we arrived here late, lunch with bread. A bread with soda? Black coffee (laughs), if it were soda! there was no more for it.

The **emotional** consequences for caregivers are related to the way they deal with the behavior of their sick family members in order to overcome their ailments or desires. Depending on the affective relationship between them and the person with HD, caregivers have feelings of helplessness, ambivalence, pain, fear, loneliness, hopelessness, sadness, overwhelm, anguish and stress. They suffer from the ambivalence of leaving their family member at home or taking him to a psychiatric home. Not all families have the option of a care center; those who can do so perceive helplessness in the face of what is happening, feeling dissatisfaction with the very fact of leaving them in a place with strangers. The feeling of sadness and especially pain arises when the caregiver sees the conditions of life of the people they love, due to a disease that has no cure, and which will continue affecting them more and more until they lead to death.

... My mother is 73 years old, and the truth is I would like to do everything to alleviate her, but I know that there is no cure for this disease, but it hurts me to see her like this, so I feel powerless.

... And seeing it reduced to that is painful… very painful… and all the dreams they had, all the illusions… right? (long silence) And day by day more deterioration, more deterioration, and they are still there… in the same fight under the same conditions.

Caregivers do not always receive specialized care to deal with their feelings, which further exacerbates conflict situations within the family and even increases the feeling of despair during daily living with a person suffering from HD.

The **physical** consequences found in this study were: fatigue, tiredness, and exhaustion from long hours, coexistence relationships, multiple activities in addition to caring for the patient, all this causing stress, a feeling of incapacity, and overload. Care in HD condition is like a battle, or a struggle. Since the disease has no cure and is degenerative, the endless strife leaves caregivers exhausted, burned out, and frustrated. In an advanced stage of the disease, a person with HD cannot perceive the tiredness and fatigue that the caregiver is going through. The burden of care is greater when the direct caregivers are the elderly, children, or adolescents. For children and adolescents, this new burden has serious implications for their own life, with a greater physical burden and responsibility that taking care of someone else involves, at a stage at which they are the ones who should be cared for by their parents or an adult. In addition, they live in constant vulnerability, in solitude, with a limited support network, and without any government support, this depriving one of one’s rights and those of the person with HD cared for by them. In addition, parents who suffer from HD will have fewer possibilities to tutor, give the company, and support their young children and adolescents. In the case of the elderly, limitations in mobility, strength, agility, and fortitude increase the challenges of caregiving.

This 89-year-old woman has many mobility problems caused by arthritis, she has deformities in her hands and feet, she cannot grasp things, nor turn a door key, she does not button her shirt, she is the caregiver of her 54-year-old daughter with HD.

Four people live in Bella Vista, the 82-year-old mother who takes care of the other three: Juanita with early-stage HD, Jimmy in a terminal state, and Manuel, who has been hearing impaired since he was a child

The physical consequences are also related to aggressive episodes. Some HD patients become aggressive, especially when they do not take the medication due to a lack of financial resources to obtain it or a lack of treatment adherence. Aggressive behaviors further destabilize the family relationship, creating a cycle of violence in which the persons with HD are violent, and their caregiver responds the same way.

What is the most difficult aspect about living together with her? Answer: She is stubborn, and rude, and she usually ignores me.. Has she hit you anytime? Answer: Oh yes, one day she grabbed her hand and tried to hit my daughter, and I got mad because she took a flip-flop to hit her. I warned her against hitting my child on her back!, and she took me by my hair like this…; of course, I hit her with a stick.

Despite all the consequences explained above, the participants express that the routines that increase their strength and creativity make the challenge of caring more bearable. Family members and caregivers undertake some strategies aimed at controlling changes in behavior, avoiding episodes of aggressiveness, ensuring that they take medications, favoring food, hygiene, and comfort, as a way of dealing with the consequences that arise when they care for a person with HD. In addition, they want people with HD to be active as long as possible; they also want to prevent them from getting lost in the street, to protect them from dangers on the street and at home, and to help to maintain their dignity. An example of this creativity is when they give the medications in food, and the person with HD does not realize it.

Caring for a person with HD is not a simple matter, and caregivers often receive negative judgments about their actions, relating them to careless abandonment and inhumanity. But many of these actions only mirror the absence of social and economic support systems and the challenges of dealing with the context surrounding people living with HD.

## Discussion

### Losing oneself

The patterns, practices, and activities that people carry out in their daily lives affirm their role in the world and their relationship with those around them. Usually, these activities or practices are carried out smoothly, without effort or care [25]. For people with HD, daily life is full of challenges, limitations, and risks that lead people to loss of autonomy, awareness, and bodily functionality. They are trapped in their own body, which no longer obeys them. Understanding this loss is a central goal of this article. The deterioration process of people with HD in the Colombian context is framed by inequitable and discontinuous access to treatment, which accelerates the disease and its complications. From this point of view, health professionals can develop institutional campaigns that help improve treatment adherence. In the context of impoverishment and social inequality, health professionals can strengthen HD support networks that empower relatives and patients to claim the right to timely and dignified treatment and follow-up of the disease [26].

### Uncertainty

Concern for their own life arises in the offspring of people with HD since they can develop the disease just as their parents did. For Strauss, said uncertainty in the chronic disease is “of up-most importance because the efficiency of social arrangements is closely related to the predictability of trajectories” [27]. Our research found differences in the decisions made by people with HD when they are informed about the disease, given that they can take measures, for example, to avoid having children and plan their lives “preparing for the worse expecting the best to happen.” Those who do not recognize their condition of being at risk also do not consider what will happen to children, their spouse, or their economic sustainability when the disease arrives.

The symptoms of HD may start at different moments of life, given that the number of CAG amino acid repeats accounts for 70% of the variability in age at disease, other genetic and environmental factors accounting for the remaining 30% [28]. Hence, the only secure option for the people at risk but have no genetic test is to avoid having children to avert increasing the number of those affected.

Many people who develop HD have anosognosia, and even though some of them may accept the deficit, they minimize its emotional or functional importance [29]. Those who recognize the onset of the symptoms have functional changes to which they initially grant little importance. As mentioned by Charmaz, upon the first changes in the chronic disease the person may think this interruption is temporary, but this is not so. The appearance of the disease is not the same for everyone, and the way each individual experiences it is different [24].

The anguish and uncertainty experienced by people at risk are related to the possibility of losing their health (losing control of their body and mind). They feel fear because their lives will be divided into before and after the disease, with some limitations in daily life and social stigma. As for alternatives to cope with uncertainty, health professionals must promote educational programs for people of all ages at risk to increase awareness of the implications of their genetic condition. Likewise, commitment is required so that more health institutions can establish and expand genetic counseling programs so that people can learn their status if they so wish [30].

### Chronicity

To understand a chronic disease, Charmaz proposes that it “arrives as an interruption, gets involved in life as an intrusion, and gets installed until it becomes an immersion, so that life is circumscribed in the disease” [24]. However, unlike a lesion that occurs acutely, HD affects conduct, and, with current therapies, the possibility does not exist of “being cured” from this condition. Thus people with HD have no way out; given the chronic nature of the disease, these people head deeper and deeper into a cave until death. In this context, palliative care is the only option since what is certain is deterioration and death. It is highly beneficial for chronic disease management that health professionals train family members in palliative care that could be executed at home. The implementation of palliative care as part of the clinical care provided to those affected in the different phases of the disease is also relevant [31].

### Loss of hope of recovery

People can live with the illness for 10–20 years until death; however, unlike other chronic diseases, like diabetes and epilepsy, which can alternate between crisis and control periods of symptoms, HD is progressive in severity; hence, those affected, and their relatives lose hope in recovery. It is difficult to accept the disease; however, specific breaking points, like a fall, an episode of choking with food or when they get lost in the street, serve to alert the family and the other affected regarding the condition of the disease. Their situation can be worse than is apparent because sometimes they have mental health concerns like anxiety, depression, or even a suicide attempt. These situations are increasingly frequent because they can no longer do what they did before. The symptoms are not temporary, and the sick person requires continuous care. The diseased people struggle to keep their jobs and responsibilities, while their functionality, ego and self-image deteriorate, all this causing them to confront reality [24]. The disease’s intermediate phase could be coped better if the people did not suffer mental or cognitive alterations, but if this phase involves mental health concerns, these impose more challenges on caregivers, increasing their burden every day [32]. Ensuring care that includes, in addition to pharmacological actions, a permanent assessment of the mental status of the patient and their caregivers, with occupational and psychological therapy to delay the severity of the symptoms, can help to give the patient better living conditions and produce fewer burdens for the caregiver [33].

### Stigma

The disease process leads to bodily changes in voice and language, loss of weight, and the choreic movements that influence the possibility of stigma, which, as Goffman indicates, is the situation of the disabled individual to be in full social acceptance [10]. The stigma worsens self-acceptance and self-image, making people more vulnerable and limiting their coping process. In this case, the discomfort is bound to signals that make the body visible, especially when people are involved in daily life and do not pay enough attention to the body's sensations. Discomfort challenges the idea of a harmonious and unproblematic body [34]. Kleinman discussed also the experience of the disease: that the body, previously a space of certainties (a non-problematic instrument), of our relationship with the world, during illness, becomes a focus of uncertainty [35]. Regarding stigma, Heath et al. found that in people with medullar lesion, loss of control over the body and the rupture with the life they had before the lesion leads to the social stigma that compromises their whole being [36]. As the changes are inevitable, it is necessary to increase the spread information on social media to generate greater compassion from the general population towards those affected. Economic contributions to organizations that support people suffering from rare diseases could favor the circulation of their experiences in the fight against stigma and raise awareness about these issues [37].

### Entering the cave

The complex sequence of changes, impairment, losses, dependency and death may correspond, according to Charmaz, to the immersion, which consists in that the disease narrows life and produces many problems when individuals start to face devastating symptoms; besides dealing with more illness, they are poorer, require reorganization of their social conditions, while not always having the means to do it. People enter a ship that takes them to profound darkness, decline, and, ultimately, to death [24]; likewise, Sontag describes that the sick body acquires new senses, challenges and is interrogated in a renovated manner by the horizon of finitude and the imminent threat of its death that accompanies it [38]. People with HD also have difficulties establishing romantic relationships, possibly due to the consequence of death that this or other diseases such as cancer or Acquired immunodeficiency syndrome (AIDS) have [38].

According to Charmaz, “the immersion into the disease means the experience of the vulnerability of their own body, confronting the dependency that is not only physical but social-economic too, the dependency that brings us closer to death” [24]. People have no way out from the *cave*; the disease transforms them, given that they are no longer what they were. This status controls them, limits, incapacitates, reduces, alienates, drowns, and isolates them from what their life was. Only a shadow remains in the memory of those who do not reach dementia; for the others, there are no memories—perhaps this brings less suffering; the exit from this cave is death. The reality of death requires the company of health professionals and support networks to accompany and prepare the farewell of the loved one who is suffering [39].

### The caregiver

As long as the affected person is dependent, caregivers look for strategies to solve their conditions [40]. According to Morin (1994) “Complexity needs strategies, used when the unexpected or the uncertain occurs, that is, since a major problem appears.”[41] (p. 63). Caring for a person with a mental state is a challenge—as expressed by Nguyen et al. [42], for the family this implies recognizing and accepting that their loved one is no longer the same, and that their transformation is not voluntary but caused by the disease. Such ideas also occur in families in the event of agitation and aggressiveness of the patient.

Research on caregivers of persons with disabilities in Bogotá (Colombia) [43] exposes the burden of care experienced by children: physical (fatigue and sleep disturbances); psychological (anxiety, stress, and depression); and social changes (behavior and socialization), as well as changes related to education since the probability of low performance in school and school dropout, increase. In our research, caregivers told about feeling stress accompanied by crying, a sense of giving up, feeling incapable, tense, and powerless when they care for a sick person with Huntington. The above are also characteristics of caregiver fatigue according to the Zarit test [44]. Cheng said that, as the disease progresses and functionality deteriorates in the patients, the pressure on the caregiver increases, with the appearance of overload in him. The caregiver of someone with dementia usually has higher comorbidities, such as a 60% greater chance of suffering from depressive or anxiety disorder [45].

The caregivers also express that they perceive insufficient support from their relatives. The work overload, inadequate support networks, and postponement of the life project itself can unleash alterations like headache, low back pain, sleep disturbances, depression, concentrating difficulty, tension, worry, and irritability. Similar to the findings of Gómez-Galindo et al. [43], it was possible for us to see how disability usually influenced the families' vulnerability, compromising their basic needs. Households with people with disabilities tend to be poorer than those with no disability [43]. Like people with disabilities, the increase in household expenses, derived from the needs that cause the disease, also concerns people with HD.

When people with HD have a physical and mental disability, the caregivers often experience increased caregiver burden and difficulty in providing efficient support in activities such as travel and transfers, personal hygiene, communication, and interaction with others. Furthermore, they have limitations for paid work, study, leisure activities, and relationships with other people due to the dependence on people who they care for. Likewise, they do not receive any payment for the care, even though this is the only job they do all day. In the context of poverty and inequity, as in the case of Colombia, health professionals are called upon to lead care programs for caregivers. They could develop strategies such as clinical and psychological assistance through telephone calls, targeting people with a digital gap, that is, those who do not have internet service [46].

## Conclusions

The lives of people with Huntington’s disease are characterized by a framework of great contextual complexity and difficult access to the services stipulated in the legislation and to health protection. The repercussions of the disease affect all spheres of life: the physical, emotional, cognitive, social, and economic drawbacks are devastating for those affected and their families.

The process of changes, losses, and impairment up to disability and death is proposed as an inverse homology of Plato's so-called *allegory of the cave*, given that the disease makes them prisoners in their own body where the reality of oneself is only shadows and memories. People with HD have special needs in relation to the general population, due to the complexity of living with the disease, and require interdisciplinary and intersectoral care, with nursing care being of central importance to offer education and programming at all levels of prevention that includes palliative care. This research seeks to provide useful knowledge that contributes to understanding the context, the person, and care, in the sense of translating the findings into benefits and advocating for the care of people with HD, their families, and the community.

## Supplementary Information

**Additional file 1:** Semi-structured interview.

**Additional file 2:** Figure the core category, subcategories, and their properties.

## Data Availability

The datasets used and analyzed during the study are available from the corresponding author upon reasonable request.

## Footnotes

1 Dysphagia is due to the esophagus contracting several times in an uncoordinated manner and with a lot of pressure after swallowing; this diffuse spasm affects the smooth muscles of the walls of the lower part of the esophagus.
