# Supplementary material for: Beyond the clinical context: the process of losing oneself living with Huntington’s disease
Source: Orphanet J Rare Dis. 2022 May 7;17:184. doi: 10.1186/s13023-022-02330-9 (PMC9077866; doi:10.1186/s13023-022-02330-9)
Supplement: Supplementary file 1 — Additional file 1: Semi-structured interview. [file 13023_2022_2330_MOESM1_ESM.docx]

**Additional file 1**

**Semi-structured interview**

**Questions:**

How did the disease spread to your family?

Describe a typical day?

Has your life changed now compared to before?

What is the activity that you do the most throughout the day?

How do you feel when you think you are at risk of developing the disease?

What are the main changes in your mood when you think about the disease?

How do you think other people perceive you?

Have you thought about the future?

How did you imagine it?

What aspects of your life have been affected by HD? why?
