# Supplementary figures and images for: Beyond the clinical context: the process of losing oneself living with Huntington’s disease
Source: Orphanet J Rare Dis. 2022 May 7;17:184. doi: 10.1186/s13023-022-02330-9 (PMC9077866; doi:10.1186/s13023-022-02330-9)

**Additional file 2**

| **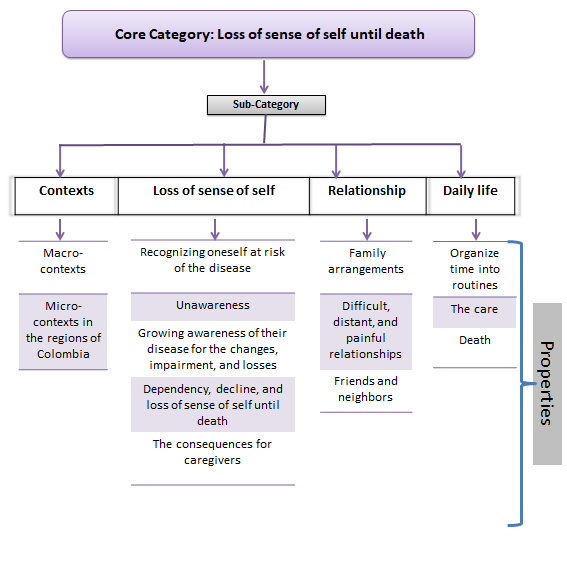** |
| --- |

**Figure** the core category, subcategories, and their properties.

Supplement: Supplementary file 2 — Additional file 2: Figure the core category, subcategories, and their properties. [file 13023_2022_2330_MOESM2_ESM.docx]
